# Factors to Consider in Gut Microbiota Interactions With the Enteric Nervous System—What You Look for Is What You Get

**DOI:** 10.1016/j.jcmgh.2026.101821

**Published:** 2026-05-27

**Authors:** Joel C. Bornstein, Rachel M. McQuade

**Affiliations:** Department of Anatomy and Physiology, University of Melbourne, Parkville, Victoria, Australia

**Keywords:** Enteric circuits, Mucosal barrier, Transcriptome

## Abstract

We review issues affecting interpretation of studies of microbiota/enteric nervous system interactions given recent transcriptomic studies and the multiple functions of the gastrointestinal tract. We highlight directions for investigation that are often overlooked in the current literature.

The gastrointestinal (GI) tract is a highly integrated sensory and regulatory organ system that coordinates digestion, barrier defense, immunity, and host-microbial interactions. The enteric nervous system (ENS) processes microbial, immune, and luminal signals and translates them into local and systemic physiological responses. It is abundantly clear that microbiota have a major impact on the ENS. This ranges from acute changes in firing of enteric neurons^1^ to alterations in neuron number (eg,^2^) and can be bidirectional as ENS changes alter microbial composition.^3^^,^^4^ Unsurprisingly, given the complexity and dynamic nature of the microbiota, the diversity of their ever-changing ecosystem, plus the many functions of the GI tract and the scale of the ENS, studies addressing mechanisms mediating this impact often require heroic experiments to isolate causative factors. Read-outs from these studies, therefore, can fall short of capturing all potential functional changes, typically concentrating on propulsion of content rather than the full panoply of neurally controlled functions. Propulsion is a core function regulated by enteric neural circuits, but mixing motor patterns and neurogenic secretion are essential for digestive processing and often overlooked. Furthermore, intestinal inflammation, mucosal barrier function, and metabolic regulation via enteroendocrine cells are all GI functions likely modulated by microbiota. These functions are mediated by some of at least 26 types of enteric neurons, defined by their identified roles, regional locations, and transcriptomes.^5^ Many studies rely on immunohistochemical profiling, but common neurochemical markers label multiple functional classes of enteric neurons; transcriptomic analysis^6^ identifies more specific, but unexploited, subtype markers. This review explores implications of these issues highlighting questions yet to be asked or answered. The message is that there are many paths to glory, and the consequences of microbiota variations may be hidden by the routes taken to study them.

## Gut Microbes Alter Enteric Neuron Behavior

Definitive evidence that commensal bacteria influence the ENS came over a decade ago with 2 key results. It was already clear that animals without microbiota (germ-free; GF) have a functioning ENS, an absolute prerequisite for survival, although GI function is impaired in GF mice. This impairment is due, in part, to alterations in the structure and function of the ENS, as seen in the ileum of 3-day-old GF mice, although the duodenal ENS resembles that of specific pathogen-free (SPF) control mice.^7^ Electrophysiological studies of intrinsic sensory neurons (IPANs) in mouse jejunum demonstrated that mucosal application of certain probiotic bacteria triggers action potentials in IPANs and increases their excitability.^1^ Both effects occur via actions in the mucosa, not via synaptic inputs to IPAN cell bodies. IPAN excitability is depressed in GF mouse colon and enhanced following colonization by SPF microbiota.^8^

## Varying the Microbiota, Germ-Free and Beyond

Most studies of microbiota–ENS interactions use a broad-brush approach, in which all bacteria within the GI tract are absent either by use of GF mice, which lack all microorganisms (including those normally in the gut), or through prolonged treatment with a cocktail of broad-spectrum antibiotics to induce a pseudo-GF state. Controls are comparisons to mice of the same strain from SPF colonies; GF mice colonized with SPF bacteria or pseudo-GF mice allowed to recover by ceasing antibiotic treatment with or without microbiota transplant from other sources. These systems allow examination of the effects of individual bacteria or defined groups of bacteria by colonization of GF gut with bacteria of choice (eg,^2^). This includes exploration of roles for microbiota related to defined disease states like Parkinson’s disease, Alzheimer’s dementia, or inflammatory bowel disease by transplanting microbiota from patients or animal models of the disease into GF or pseudo-GF animals. These approaches allow conclusions about whether microbiota is necessary and provide tools for analysis of potential mediators but fall short of identifying how alterations in microbiota due to lifestyle changes like diet or exercise affect ENS function. Importantly, they lack the temporal resolution to determine the dynamic interplay between microbiota and host physiology.

## The Importance of Host Physiology

Sex is a key factor in determining microbial/ENS interactions, as altering neonatal microbial colonization of the murine GI tract produces sex-dependent changes in ENS structure, neuronal excitability, and synaptic function in young adults.^9^ More generally, host lifecycle is an often unexplored parameter for studies of the physiology of microbiota–ENS interactions. The development, maturation, maintenance, and senescence of the ENS all present different challenges, as do effects of weaning (obligatory changes in diet), puberty, pregnancy, the reproductive cycle (menstrual/estrus cycle), and menopause. Age may be critical for studies relating to ENS development because mice, the commonly preferred experimental model, are born with an immature ENS that is exposed to colonizing microbiota during neonatal maturation. In contrast, the human ENS reaches similar maturity in utero, so microbial colonization occurs weeks after initial ENS maturation. How this fundamental difference affects generalizations between species is unknown.

The microbiota has roles throughout development. Although it is usually assumed that the GI tract of a fetus is sterile, maternal microbiota affect ENS development even prior to birth.^10^ Bacterially produced formylated peptides from the maternal gut presumably mediate effects in the fetal ENS, as they are not seen in pups lacking formylated peptide receptors 1 and 2 in enteric neurons and precursors. Perhaps operating in parallel with formylated peptides, effects of modifying colonization of the neonatal mouse gut with vancomycin are partly mediated by mucosal serotonin (5-hydroxytryptamine [5-HT]) both during treatment^11^ and 4 weeks later.^9^ Other bacterially derived mediators are also implicated, including short-chain fatty acids (SCFAs), secondary bile salts,^12^ and cell wall components. Similarly, perturbing the microbiota without producing a pseudo-GF state around weaning alters ENS structure and function.^13^ Comparative studies at other life stages and with similar perturbations are lacking but essential.

The days needed to change the composition of the microbiota either environmentally (diet, antibiotics, fecal microbiota transplant) or colonization of GF gut necessarily limit temporal analysis of microbiota–ENS interactions. This impinges on interpretation, as time courses of effects in other elements of the system are much faster. Enteric neurons respond to physiological stimuli in milliseconds and exhibit plastic changes in neurochemistry,^14^ synaptic function,^15^ and firing properties^16^ within hours. Probiotic bacteria applied to the epithelium trigger action potentials in IPANs within 8 seconds and increase their excitability along with motility within 15 minutes.^1^^,^^17^ Small molecule mediators like 5-HT, adenosine triphosphate, or low pH applied to the mucosa trigger responses within 800 milliseconds.^18^ IPANs exhibit prolonged increases in excitability within 2 hours of exposure to bacterial exotoxins like cholera toxin (CT) and toxin A of *Clostridium difficile* (a *Clostridium difficile* exotoxin) and in response to acute inflammation.^16^ These increases persist for several hours and for days after inflammation, despite all overt inflammation having been resolved.^19^ The rapid change in excitability of input neurons to enteric neural circuits implies that these circuits, which are continuously physiologically active, change their behavior in response to variations in bacterially derived mediators prior to currently measured changes in microbial composition. As motility and electrolyte secretion are critical for nutrient digestion and absorption, changes in enteric neural circuits will alter the ecology of the microbiota while it is being modified by externally imposed stimuli. A further complication comes from recent findings that IPANs directly transmit to extrinsic nerve endings in the GI wall^20^^,^^21^ and so can affect appetite, satiety, and interoception during microbial changes. Monitoring local changes in microbial composition over timeframes comparable to reactivity of other components of the GI tract is essential to fully understand the contributions of microbiota to ENS function. A first step may be to engineer bacteria of interest to produce fluorescent markers so that their local abundances can be monitored in vivo via abdominal windows like those developed to monitor enteric neural activity using live cell imaging in situ.^22^

## Access to Enteric Nervous System Receptors for Microbes, Their Metabolites, and Secondary Mediators Is Crucial

**Figure 1:**
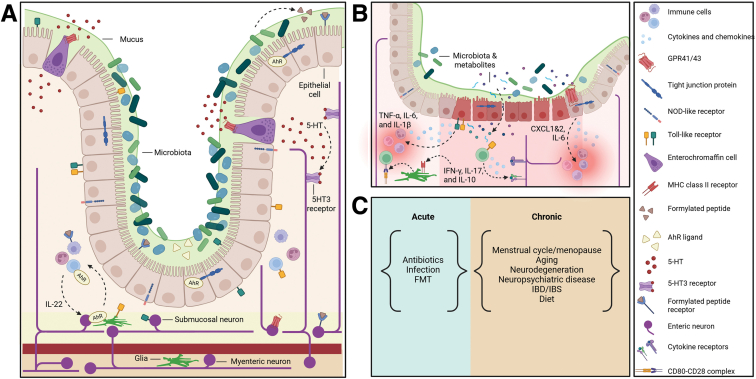
**Factors affecting barrier-dependent access of microbial signals to the enteric nervous system.** (*A*) Under physiological conditions, the intestinal epithelium, mucus layer, and immune surveillance systems spatially restrict luminal microbiota from direct contact with the ENS. Microbial metabolites diffuse to and across the epithelium and engage epithelial receptors (eg, aryl hydrocarbon receptor [AhR]), stimulating enteroendocrine cells to release mediators such as 5-HT, which acts on 5-HT3 receptors on adjacent neuronal terminals to indirectly modulate enteric neuronal activity. Immune-derived cytokines (eg, IL-22) reinforce barrier integrity and epithelial renewal. Bacterially derived formylated peptides may also signal through formyl peptide receptors (FPR1/2) expressed on epithelial cells and enteric neurons or their precursors, representing another route by which microbiota-derived molecules influence ENS development and function. In this state, communication between microbiota and the ENS is predominantly indirect, regulated, and compartmentalized. (*B*) During epithelial injury or inflammatory challenge, disruption of tight junctions and mucus architecture increases luminal access to the mucosa and subepithelial compartments. Importantly, the resilience of this barrier is metabolically conditioned. Microbially derived SCFAs, generated through dietary fiber fermentation, promote tight junction integrity and mucus production and signal via G-protein–coupled receptors (eg, GPR41/43) on epithelial and immune cells to regulate chemokine and cytokine output. Reduced fiber intake lowers SCFA availability, rendering the epithelium more susceptible to pathogen encroachment and amplifying inflammatory cascades. Under these conditions, epithelial and immune cells release proinflammatory cytokines and chemokines (illustrated as *blue dots*; eg, tumor necrosis factor [TNF]-α, IL-6, IL-10, IL-17, IFN-γ, IL-1β, C-C motif chemokine ligand [CXCL]1/2) that recruit and activate immune populations and alter epithelial and neuronal signaling. Inflammatory conditions can also induce the expression of MHC-II and CD80/86 on enteric glia, enabling them to act as nonconventional antigen-presenting cells that directly activate CD4+ T-lymphocytes via the CD28 receptor. These mediators, together with increased epithelial permeability, allow microbial products greater access to deeper tissue layers, increasing the probability of heightened or direct signaling to enteric neurons and glia. (*C*) A wide range of conditions can affect ENS–microbiota communication either acutely or chronically. Their various effects reflect a spatially and temporally dynamic gradient, in which structural barrier integrity dictates whether signaling remains metabolite-mediated and indirect or becomes inflammation-amplified and permissive to deeper tissue exposure. Myenteric and submucosal plexuses are depicted schematically for conceptual purposes and do not represent the full diversity, identity, or relative abundance of neuronal subtypes within these networks. Similarly, the mediators illustrated are representative examples intended to highlight key signaling pathways rather than a comprehensive catalogue of all microbiota-derived, epithelial, immune, or neuronal mediators involved in ENS–microbiota communication. FMT, fecal microbiota transplant; IBD, inflammatory bowel disease; IBS, irritable bowel syndrome.

An often-underestimated issue is whether microbes or their products reach their receptors on enteric neurons, glia, and other cell types that they modulate (Figure 1*A* and *B*). This is highlighted by how CT produces hypersecretion and hypermotility in the duodenum/jejunum.^16^ Although secretory enterocytes, enterochromaffin (EC) cells and IPANs all express CT receptors (GM-1 gangliosides), the geometry of the small intestinal mucosa means that CT only reaches EC cells, with secretory enterocytes located deep in the crypts and IPAN terminals protected by the mucosal barrier. CT binds to EC cells at the villus tips, triggering 5-HT release that excites IPAN terminals. IPANs and secretomotor neurons become hyperexcitable, thereby massively enhancing secretomotor and propulsive motor activity. Similar issues apply to more physiological, microbial mediators, which are typically sequestered from the ENS by the mucosal barrier (Figure 1*A*) and may only reach their targets after pathologic disruption of the barrier (Figure 1*B*).

The intestinal barrier is the critical interface through which microbially derived signals must pass to reach enteric neurons or the blood stream. This is not passive, but a dynamic regulator that shapes the identity, magnitude, and timing of signals reaching enteric neurons and neighboring cells. Tight junction complexes regulate paracellular permeability, modulating the access of microbial metabolites, microbe-associated molecular patterns, and pathogen-associated molecular patterns to subepithelial compartments, whereas overlying mucus maintains spatial segregation and establishes gradients shaping epithelial exposure to luminal communities. Critically, epithelial cells are environmental sensors, translating microbial and dietary signals into cytokines and neuroactive mediators that influence immune and neuronal networks.

Intestinal epithelial and enteroendocrine cells express an array of pattern-recognition and metabolite-sensing receptors involved in microbial sensing and signal transduction; many are also expressed by enteric neurons and glia (Figure 1*A*). Pattern recognition receptors, including Toll-like receptors and NOD-like receptors, detect microbial-associated molecular patterns, triggering cytokine and chemokine release that shapes subepithelial immune activity and indirectly influence enteric neuronal excitability.^23^, ^24^, ^25^ SCFAs acting at FFAR2/3 and GPR109A, as well as intracellular histone deacetylase–dependent pathways, can modulate epithelial barrier function and stimulate enteroendocrine secretion of 5-HT and peptide hormones) with established neuromodulatory effects.^26^ Microbial tryptophan metabolites activate the aryl hydrocarbon receptor, promoting epithelial transcriptional programs that influence barrier integrity and interleukin (IL)-22–dependent immune-neuronal crosstalk.^27^ The same receptors are expressed by enteric neurons,^28^ and distinguishing their effects from alterations in the barrier is a challenge requiring selective deletion of the receptors from each cell type (ie, from enterocytes, neurons, glia, and immune cells). Similar approaches also apply to other widely expressed receptors (Figure 1*A* and *B*).

## Correlating Changes in Gastrointestinal Function With Changes in the Enteric Nervous System

Many studies monitor physiological changes produced by altered microbiota by measuring alterations in gut motility, notably intestinal transit, and correlate this with altered numbers of enteric neurons and/or their neurochemistry.^10^^,^^28^ There are, however, several other major GI functions regulated by the ENS, including mixing, transmucosal water and electrolyte transport, regulation of the mucosal barrier, GI immune function, and neuroendocrine functions that regulate host metabolism and appetite. These functions interact closely with motor activity and should be assessed in parallel with motility, as propulsion is not a surrogate, even for mixing, and stool water content is not an adequate measure of secretion because net fluid absorption is also influenced by propulsion and mixing. Furthermore, restoration of normal neuronal density due to neurogenesis after a neuropathic insult does not always restore intestinal motility,^29^ perhaps because loss of neurons disrupts the complex network of axons and synapses that control motility and other GI functions, and this network must be reestablished before normal functions are fully restored.^30^ The network may never be fully re-established either because of inadequate pathfinding or inappropriate synaptogenesis. Regrowth of axons and synapses is guided by enteric glia,^31^ whose regulation by the microbiota is not as extensively studied despite strong evidence that it happens.^2^^,^^32^

Enteric glia are important both because they have key roles in many GI functions, including the intestinal barrier, and play a dynamic part in enteric neural circuit activity (for review^33^) and because some are precursors for adult neurogenesis.^34^^,^^35^ Indeed, glia are targets for many mediators resulting from microbial metabolism and from immune responses to microbes and their metabolites (Figure 1*A* and *B*). Transcriptomic studies have identified several subtypes of enteric glia^36^ supporting anatomic and physiological studies (eg,^37^^,^^38^). How the microbiota affect these glial subtypes remains uncertain, and how glia actually communicate with neurons is an area of active research interest.

The identities of the neurons affected are also a significant unknown. Recent transcriptomic studies show that standard methods of identifying neuronal subtypes, notably immunohistochemical labeling for choline acetyltransferase (cholinergic) and/or neuronal nitric oxide synthase (nNOS), cannot discriminate specific classes of enteric neurons. For example, a very recent, and elegant, meta-analysis of several transcriptomic studies^6^ identified 9 classes of neurons that express nNOS, raising the question as to which of these are lost after antibiotic cocktail treatment,^9^^,^^11^^,^^13^ and what are their specific functions. Furthermore, do the different subtypes reappear in their normal proportions when the gut is recolonized, and how is this regulated? Similar issues apply to studies reporting changes in cholinergic neurons. A starting point for addressing such issues is to use subtype discriminating markers identified in transcriptome studies either in multiplex immunofluorescence studies or by targeted reporter expression as in^39^^,^^40^ in place of the broad markers that have been standard. Note, anatomic analysis of targeted reporters will provide answers to questions about circuit rearrangements, regeneration, and synaptogenesis not addressed in current studies. That this is important is shown by the finding that some cholinergic neurons make synapses with nNOS inhibitory motor neurons, so changes in their numbers will alter inhibitory drive to the muscle independently of changes in nNOS neurons. These questions are highly relevant to understanding how the microbiota are coupled to function as cholinergic neurons include secretomotor/vasodilator neurons, IPANs, excitatory motor neurons, and several classes of interneurons with distinct pathways being excited by different stimuli.^41^ As yet, transcriptomic analyses have not discriminated candidate cholinergic interneurons from myenteric IPANs, although submucosal IPANs can be distinguished from cholinergic secretomotor/vasodilator neurons.^39^ Furthermore, some cholinergic interneurons are immunoreactive for nNOS. Similarly, single-nucleus RNA sequencing analyses have yet to identify other known classes of interneurons, notably ascending cholinergic interneurons and anally projecting 5-HT/cholinergic interneurons, although, the latter, identified immunohistochemically, are altered in a sex-dependent fashion by neonatal perturbation of microbial colonization.^9^

## Regional Differences in Susceptibility and Functions Are Core Issues for the Enteric Nervous System and Microbiota

Most GI microbiota are found in the large intestine, and there are major differences in bacterial species and properties between the colonic microbiota and more rostral regions. Similarly, enteric neurons regulate many interacting functions that vary in physiological significance between regions,^5^ which is reflected in transcriptomic differences. Thus, a complete analysis of the effects of the microbiota requires investigation of each of these functions and several regions (see for example,^40^^,^^42^ which began this massive task). There are clear differences in susceptibility to microbial perturbation between the small and large bowel and even between the duodenum and ileum.^7^^,^^11^^,^^42^^,^^43^ The duodenum represents a special case, because its ENS retains its normal structure and function in GF mice^7^ and in neonatal mice with perturbed microbial colonization.^11^ By contrast, jejunum, ileum, and colon show similar effects following microbial manipulation, although mediated via distinctly different mechanisms. For example, recovery from effects of an antibiotic cocktail is mediated by T cells in jejunum but not colon.^2^ Similarly, resident GI microbes regulate mucosal 5-HT synthesis in colon but not the small intestine.^44^^,^^45^

It is also important to consider the submucosal plexus, which is central to regulation of water and electrolyte movement across the mucosa and, hence, to secretory diarrhea.^16^ Submucosal IPANs are present in the mouse duodenum but not in the colon^39^; thus, the region studied is highly relevant to interpretation.

Further complexity comes from significant evidence that the composition of the microbiota varies according to the properties of the ENS. On a slow time scale, mice expressing a mutated synaptic protein in enteric (and central) neurons have altered fecal microbiota^3^ and mucus profiles.^4^ More dynamically, increased secretion produces diarrhea, thereby altering microbial composition, whereas increased permeability of the mucosal barrier allows bacterial (and bacterial metabolic products) access to the ENS that would normally be prevented (Figure 1*B*). Determining the time dynamics of microbial changes, as discussed above, will be essential to resolve these and related issues. Diet and other environmental factors can also modify microbial composition and aspects of ENS function, but which drives which is uncertain, especially as diet can alter the mucosal barrier.

One important time–course issue is that changes in enteric neuron function produced by an initial perturbation, say by antibiotic cocktail treatment, likely begin within hours, well before the 10 to 14 days of the full treatment regime. Thus, outcomes reflect both overall loss of microbes alongside altered neural activity during the treatment. This may not matter to interpretation of the broadbrush antibiotic cocktail or GF studies but does not reflect normal physiology or modified microbiota due to lifestyle or medical treatment.

## Role of Diet

Diet represents a major upstream determinant of microbiota-ENS signaling, chronically shaping both microbial composition and the metabolic landscape of the lumen (Figure 1*C*). SCFAs regulate epithelial tight junction expression and mucus production^46^ and activate G-protein-coupled receptors (eg, GPR41/43) expressed on epithelial and immune cells, triggering rapid chemokine and cytokine release (Figure 1*B*).^47^ Fiber depletion reduces SCFA availability, thins the mucus layer, and increases epithelial access for the mucosal pathogen, *Citrobacter rodentium*, resulting in lethal colitis.^48^

Recent work^41^ highlights that distinct nutrients activate spatially and functionally segregated neuronal populations, supporting the concept of nutrient-specific coding within the ENS. Thus, dietary inputs acutely influence enteric network dynamics, with microbiota and derived metabolites potentially acting as dynamic intermediates in signaling nutrient levels and dietary composition.^28^ Signals emerging from epithelial and dietary–microbial interactions are embedded within a broader neuroimmune network in which mucosal immune cells are critical intermediaries.^2^ Microbiota-dependent shaping of mucosal T cells introduces another layer of regulation. T cells mediate microbiota-induced changes in ENS structure^2^ and function and may contribute to recovery following neurotoxic injury.^29^ Diet-induced or microbial-driven shifts in T cell subsets alter cytokine profiles within the lamina propria, and cytokines such as interferon-γ, IL-17, and IL-10 modulate of neuronal excitability and synaptic transmission. Importantly, these immune-mediated effects have different time courses to acute metabolite signaling, complicating interpretation of ENS changes following dietary intervention, antibiotic treatment, or colonization paradigms. A key unresolved question is therefore whether observed neuronal transcriptional shifts reflect direct sensing of microbial metabolites, secondary responses to immune activation, or adaptive remodeling following barrier perturbation. Resolving this hierarchy of signaling events will require longitudinal and cell-type–specific functional studies that integrate physiology with multiomic profiling.

## Final Thoughts

Although current studies reveal a wealth of information, they typically do not identify mechanisms and issues related to physiological or lifestyle-related variations in microbial composition. The key questions are no longer whether microbes influence the ENS and its development, but rather which specific enteric neurons and circuits are affected, how this reshapes integrated GI functions over time, and how microbiota-induced changes in the ENS or mucosal barrier feedback to affect the resident microbial community and modulate this dynamic balance.
